# Modulation of autonomic responses to cognitive tasks under acute mental stress

**DOI:** 10.1038/s41598-025-34921-4

**Published:** 2026-01-08

**Authors:** Stefania Coelli, Matteo De Tommaso, Pierluigi Reali, Rossana Actis-Grosso, Anna M. Bianchi

**Affiliations:** 1 Department of Electronic, Information and Bioengineering, Politecnico di Milano, Piazza Leonardo da Vinci 32, 20133 Milan, Italy; 2 Department of Psychology, Università degli Studi Milano – Bicocca, Milan, Italy; 3 Milan Centre for Neuroscience, Milan, Italy; 4 Fondazione IRCCS Ca’ Granda Ospedale Maggiore Policlinico, Milan, Italy

**Keywords:** Physiological measurement, Heart rate variability, Electrodermal activity, Respiration, Mental stress, Neuroscience, Physiology

## Abstract

Acute mental stress activates the autonomic nervous system (ANS), modulating physiological parameters. To assess the ANS response, we collected multimodal physiological signals, including electrocardiogram (ECG), electrodermal activity (EDA), and respiratory activity from healthy participants. The experimental protocol was designed to induce a high stress level in one group (STRESS) and low stress in the other (CONTROL), undergoing the same cognitive tasks. Heart rate variability (HRV) indices, parameters from respiratory activity and EDA were computed and analyzed. First, the effect of the proposed stress manipulation on the ANS was assessed, showing that linear HRV and respiratory parameters significantly changed during cognitive tasks with respect to rest in both the groups, mainly when respiration activity was integrated in the analysis. Nonlinear HRV parameters and EDA-based indices presented more task-specific modulations. Significant differences among groups were found only for the mean RR interval and the EDA-derived parameters. Additionally, Random Forest models were trained, and feature importance was assessed through Shapley values. Results identified the amplitude of the phasic EDA component, respiratory sinus arrhythmia (RSA), HRV sample entropy, and mean breathing period as the features most clearly differentiating cognitive tasks from rest, highlighting the importance of a multimodal assessment of acute stress.

## Introduction

In its negative connotation, psychological stress (i.e., distress) is caused by the disruption of balance between perceived cognitive and emotional load induced by external stimuli, and the individual’s ability to cope with them^1,2^. This condition can be transitory (acute phase) or can persist for longer periods (chronic condition), impacting the subject’s quality of life. Specifically, acute stress episodes may temporarily affect human mood, attention, and engagement; potentially reducing work- and study-related efficiency. A sustained exposure to stress can also be a trigger for cardiovascular events^3^. The production of cortisol and other biological mediators is the result of an active process aimed at maintaining stability, referred to as ‘Allostasis’, but when the stability is altered, the body is forced to a new equilibrium causing an ‘Allostatic load’ that can be harmful^4^.

Thus, from a physiological point of view, events perceived as stressful induce a complex sequence of responses, comprising an interplay between the central and autonomic nervous systems (ANS) and endocrine regulation^5,6^, often measured through cortisol concentration^7^. This mechanism has been shown, by many laboratory-based studies, to modulate measurable ANS-related parameters^2,8–11^, principally in terms of heart rate variability (HRV). HRV is a recognized marker of cardiovascular health^12^, which can be derived from the electrocardiogram (ECG) and other related signals such as photoplethysmography (PPG). Specifically, the ANS works with other physiological systems to regulate the heart rhythm, increasing and decreasing the heart rate (HR) through the activation of the parasympathetic and sympathetic branches of the nervous system. In stressful conditions, it has been demonstrated that a predominance of sympathetic activity is reflected in an increase of HR and a decrease of the variability, in both acute and chronic conditions^2,13–15^. Other studies also evaluated responses to stressors and emotional stimuli using respiratory parameters^16–18^, or pointed out the usefulness of integrating the respiratory information to refine the HRV analysis^8,19^. In the latter case, the possibility to disentangle the respiratory contribution from the HRV signal has been shown to improve the estimation of frequency-domain HRV parameters, particularly of the respiratory sinus arrhythmia (RSA)^20^, also recently named Respiratory Heart Rate Variability (RespHRV) to reduce the potential pathological connotation^21^. RSA quantifies the influence of respiration on oscillations of the HRV signal, which is associated with vagal control and expected to decrease under stress.

Another set of parameters widely used in the affective research field is derived from the electrodermal activity (EDA) signal (i.e., Skin Conductance response), which has been demonstrated to respond to emotional stimuli^22,23^, mental load^24,25^, and stress manipulation protocols in multimodal frameworks^9,26,27^.

In recent years, the possibility to monitor vital signs, using wearable and smart technologies, has increased research interest in understanding which physiological parameters better detect acute stress events, to potentially prevent chronic conditions^28–30^. Even so, how the ANS responds to stress has still not been fully characterized, and research findings are often conflicting. In fact, psychological stress is a very complex condition composed of several factors belonging to social, cognitive, physical, and psychological domains^31^. In this context, the first objective of the study was to evaluate the effectiveness of different physiological parameters in characterizing ANS responses to acute mental stress. A second aim was to assess the effects of varying degrees of acute mental stress by comparing the ANS responses to subsequent cognitive tasks in two groups of individuals after different exposure to stress (i.e., a Stress and a Control group). To this aim, short-term (< 5 min) linear and nonlinear HRV indices from the ECG signal, time-domain parameters from respiratory activity, and features from EDA were analyzed. Finally, we investigated the relationship between physiological responses, psychometric variables, and biochemical parameters (i.e., cortisol concentration).

Specifically, our study was based on a randomized acute stress manipulation protocol in which the Montreal imaging stress task (MIST)^32^ was used to induce acute stress in half of the study population (31 participants), while the other half (31 participants) performed a modified, less-challenging MIST version as a control condition. After this stress-inducing task, two cognitive assignments were also performed: the mixed gambling task (MGT) and a spatial attention task based on visual search (VS), which were administered equally to all participants. Physiological signals (i.e., ECG, respiration, and EDA) were continuously acquired, while psychometric assessment and biochemical samples to measure cortisol release were collected at specific time points during the procedure. By taking advantage of a multidisciplinary approach, the results of our study yield comprehensive insights into characterizing the effects of acute stress on physiological responses in healthy adults.

## Materials and methods

### Participants

The described study was compliant with the Declaration of Helsinki and approved by the Ethics Committee of Politecnico di Milano (opinion n°12/2024), where the data collection took place.

A total of 62 participants (30 male, 32 female) with an age between 18 and 40 years (mean age 30.2, SD 6.9 years) were recruited for the experiment. Inclusion criteria comprise the absence of any cardiovascular, neurological, or psychiatric pathologies. Participants were recruited through an online advertisement of a specialized recruiting agency, and monetary compensation was agreed upon acceptance. Volunteers received instructions about the experiment and all the documentation by email and were asked to refrain from caffeine consumption and intense physical activities for 24 h preceding the experiment. All subjects were Italian speakers.

### Experimental procedure

On arrival, participants were asked to read and sign the informed consent form before the biomedical signal recording equipment was set up. They sat in a comfortable chair in front of a 27” computer screen at a distance of approximately 80 cm, and an Italian keyboard was given to perform the assignments. Participants collected the first salivary sample. Subsequently, a general description of the experiment was shown on the computer screen before the procedure started. To estimate the initial stress level, two self-assessment questionnaires (Italian versions) were digitally filled out, namely the Profile of Mood States (POMS) scale and Perceived Stress Scale (PSS) answering to each question with a value between 1 and 5. Finally, participants were asked to identify their level of stress from 0 to 100 using the Subjective Units of Distress Scale (SUDS), which measures the level of perceived stress at a specific time.

**Fig. 1:**
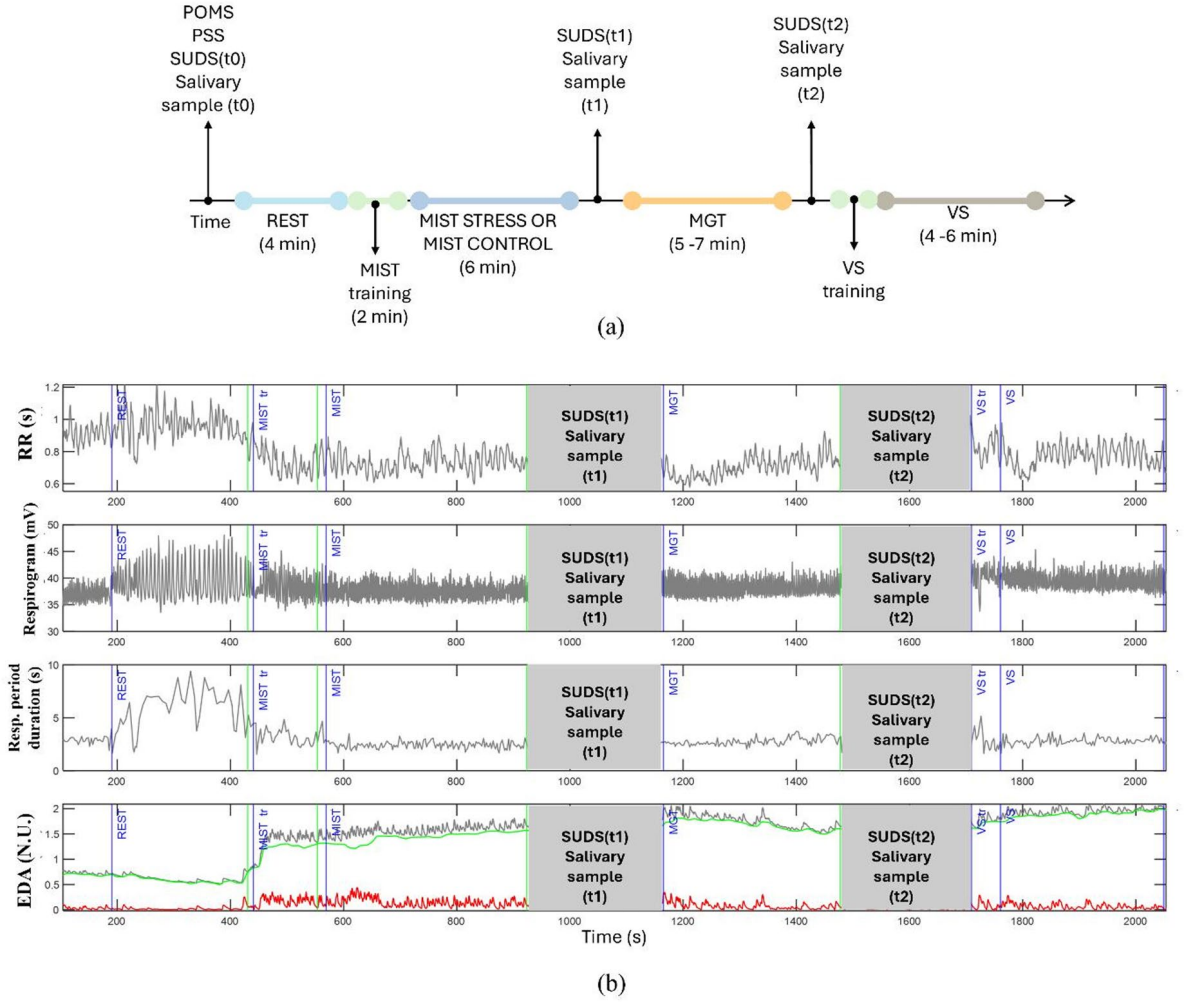
(**a**) Experimental protocol. (**b**) Example of the time series of interest from a participant. The first row shows the corrected tachogram (RR series), the second row displays the respirogram derived sampling the respiration signal using the RR series. The third row displays the respiratory period duration series derived from the respiration signal. Finally, the EDA signal in gray, its tonic component in green and the phasic component in red are displayed in the last row. Gray areas represent the time periods in which the cortisol and SUDS were collected, thus artifacts can be present in these phases, which were discharged. Blue lines indicate the start of each phase, green lines their end.

Data collection started with a resting phase of four minutes (REST), during which a gray fixation cross was displayed in the center of the screen on a black background. To provide a baseline for physiological measurements, participants were asked to remain still, with eyes open, and were invited to relax. To study the influence of the previous task on the subsequent one, task order was fixed for all participants, with the sequence depicted in Fig. 1(a): after the REST phase, the Montreal Imaging Stress Task (MIST)^32^ was implemented to induce higher stress levels in half of the participants (STRESS group) and to be less stressful in the other half (Control group); then, the Mixed Gambling Task (MGT) was presented after three minutes of break and was followed by a spatial attention exercise, specifically the visual search (VS) task. All tasks were preceded by written instructions, MIST and VS also by a short training phase. Saliva samples and SUDS responses were re-collected after MIST and MGT. The protocol was implemented and managed using MATLAB and Psychtoolbox-3 (available at https://www.psychtoolbox.net/).

Participants were randomly assigned to the CONTROL group (31 participants, 18 females), which underwent the less-challenging MIST task, and to the experimental one (STRESS, 31 participants, 14 females) that performed the demanding MIST task with additional stressing factors.

#### Montreal imaging stress task (MIST)

The MIST task is a digital protocol proposed by Dedovic^32^ designed to induce psychological stress in participants by asking them to solve arithmetic operations. In this study, two MIST protocols were implemented: an experimental one, assigned to the STRESS group, aimed at inducing sustained mental stress through demanding cognitive efforts; an easier one, administered to the CONTROL group. Specifically, in the experimental version, following the description and instructions provided by the original proposing study and subsequent implementations^33^, participants were asked to quickly solve several arithmetic operations, with 5 levels of difficulty (randomly mixed), under time pressure, and social pressure due to the presence of the experimenter next to the participant. Moreover, a bar showing their progress (i.e., increasing with correct answers and decreasing with each error) was constantly shown on the screen. Mistakes were underlined by unpleasant sounds. The easier version of the MIST, instead, proposed the same arithmetic operations, with mixed difficulty levels, but without any time constraint displayed on the screen or social pressure, and correct answers were accompanied by a pleasant sound. For both conditions, a short training phase was performed to calibrate the time given for answering MIST arithmetic operations and allow participants to practice before the actual task. Based on guidance from the literature^32,33^, during the training phase, which was designed to last two minutes, participants performed 21 trials on average (SD = 1.8), depending on their reaction times, sufficient to estimate their mean reaction times and to calibrate initial difficulty. The time constraint was set to minimize unnecessary task exposure prior to stress induction. The experimental MIST took six minutes during which the initial calibration parameters were adaptively updated to match participants’ performance and maintain the desired level of difficulty.

#### Mixed gambling task (MGT)

During the MGT, task participants were asked to accept or refuse bet proposals, each involving a specific number of virtual points as potential gain or loss^34,35^. Participants were informed that, at the end of the protocol, among the accepted bets, five would be randomly selected and the outcome would have been determined by chance in order to calculate the final ‘bonus’ to be added to the agreed payment. MGT phase lasted between 5 and 7 minutes, and the number of bet proposals to be evaluated was fixed.

#### Visual search (VS)

The VS task was performed after a short practice phase. The stimuli consisted of one L and one T (1.8° × 1.8°), presented simultaneously and spaced 180° apart on an imaginary circle with a 6° radius, centered on the screen, and participants were instructed to detect the T (target). The two letters appeared randomly tilted to the left or to the right, and participants had to indicate the direction of the tilted T as quickly as possible using the left/right arrow keys on the keyboard. A fixation cross, inscribed in a circle with 0.5° diameter, was displayed at the center of the screen. A too slow or incorrect target identification was accompanied by an unpleasant sound for both the groups. For each stimulus, reaction time and accuracy were recorded. A fixed number of trials was presented to participants, and the VS task lasted between 4 and 6 min.

### Data recordings

Physiological data were collected at the B3 Lab, Politecnico di Milano, Italy. EDA, ECG, PPG, and respiratory signals were simultaneously recorded using the ProComp Infiniti (Thought Technology, Canada), an 8-channel polygraph. ECG and respiratory signals were sampled at 2048 Hz, while PPG and EDA at 256 Hz. The EDA sensors were attached to the palm side of the annular and middle finger on the left hand for all participants, since the PPG sensor was attached to the index finger. Participants were asked to use their right hand to solve the tasks using the keyboard. ECG was acquired using three disposable electrodes applied in lead-I configuration. The respiratory signal was measured using a chest strap equipped with a resistive sensor positioned at the level of the sternum. To minimize artifacts and improve signal quality, participants were asked to remain still throughout the experiment. In this study, the PPG signal was not analyzed, since we focused on the ECG signal to extract HR-related parameters. Salivary samples were collected using Salivette Cortisol^®^ (Sarsted) and analyzed by an external laboratory.

### Physiological data analysis

#### Signal pre-processing

The Pan-Tompkins algorithm^36^ was used to identify R peaks in the ECG to obtain the RR series (tachogram), and results were manually checked to correct misdetections and remove ectopic beats through an in-house MATLAB graphical interface. The identified peaks were used to compute the RR series, i.e., the series of the time distances between consecutive heartbeats^12^. The respiratory signal was low-pass filtered at 10 Hz with a zero-phase FIR filter using a Kaiser window with 7426 coefficients. A series synchronous with the RR signal, called respirogram, was extracted by sampling the amplitude of preprocessed respiratory signal in correspondence with each identified R peak^12,37–40^.

The low-pass filtered respiratory signal was also further processed to derive the series of respiratory period durations, that is the series of the time distances between consecutive breathing actions. Specifically, following and adapting the processing pipeline proposed in the literature^41^, it was downsampled to 64 Hz and further filtered between 0.05 Hz and 1 Hz using a Butterworth filter (order 4, zero-phase implementation) before detecting the positive peaks of the waveforms using the MATLAB function ‘findpeaks’. A minimum distance of 1.5 s, corresponding to a maximum respiratory rate of 40 respirations per minute, and a prominence of 0.2 were imposed. Results of the procedure were visually checked to ensure the correct detection of breathing actions.

The EDA signal was low-pass filtered at 2.5 Hz using a zero-phase FIR filter (Kaiser window, 586 coefficients), downsampled to 16 Hz, and normalized by applying the z-score transformation. The open-source Ledalab toolbox^22^ was used to decompose the signal into its tonic and phasic components. Specifically, the first reflects the slow-changing baseline level of skin conductance over time (i.e., Skin Conductance level – SCL), while the second contains rapid changes associated with transient activations, called Skin Conductance Responses (SCR), represented by the phasic component.

Data from two participants belonging to the control group were removed due to failed recording in one case and low-quality signal in the other, resulting in 29 subjects in the CONTROL group and 31 in the STRESS group. An example of the analyzed signals is displayed in (Fig. 1b) for one participant.

#### Linear time-domain and frequency-domain HRV parameters

Time-domain and frequency-domain short-term (< 5 min) HRV parameters^14^ were extracted from the HRV signals (RR series and related respirogram), considering the central four minutes of recording for each protocol phase. In the time domain, the mean RR (meanRR [ms]), RR standard deviation (stdRR [ms]), and root mean square of successive R peak differences (RMSSD [ms]) were computed.

Concerning the frequency domain, the power spectral density (PSD) was estimated using the Autoregressive (AR) modelling approach^42,43^, both in a univariate and in a bivariate fashion. Specifically, linear trend was removed from individuals’ HRV signals to reduce spectral contribution of the lowest frequencies and highlight faster oscillations. For each segment, first an AR model of order p based on the expression in Eq. (1).

1 $$\:y\left(n\right)=-{\sum\:}_{i=1}^{p}{a}_{i}y\left(n-i\right)+u\left(n\right)$$

where $\:y\left(n\right)$ is the HRV signal at sample $\:n$, $\:y(n-i)$ indicates previous samples, $\:{a}_{i}$are the coefficients of the AR model, and $\:u\left(n\right)$ is the white noise having zero-mean and variance $\:{\sigma\:}^{2}$. The optimal order p of the AR model was automatically selected between 7 and 15 using the Akaike information criterion (AIC)^12,20^ for each processed segment and the model was estimated using the Yule–Walker formulation. Once the AR(p) model is estimated, the transfer function of the model is given by Eq. (2).

2 $$\:H\left(z\right)=\frac{1}{(1+{\sum\:}_{i=1}^{p}{a}_{i}{z}^{-1})}=\frac{1}{A\left(z\right)}$$

3 $$\:PSD\left(f\right)={\left.\frac{T{\sigma\:}^{2}}{A\left(z\right)A\left({z}^{-1}\right)}\right|\:\:}_{z={e}^{2\pi\:jfT}}$$

From which the representation in the frequency domain, the PSD(f), can be estimated as in Eq. (3).

with T being the sampling period, corresponding to the average duration of the RR intervals in the considered segment^20,39^.

From the obtained PSD, powers in LF (0.04–0.15 Hz) and HF (0.15–0.4 Hz) bands were estimated as the sum of the individual contributions of the poles that fall in the frequency range of each band. Moreover, the LF/HF ratio and the normalized LF and HF (normalized units, n.u.) powers were also extracted as relative values to the total power minus the very low frequency component^12^.

**Fig. 2:**
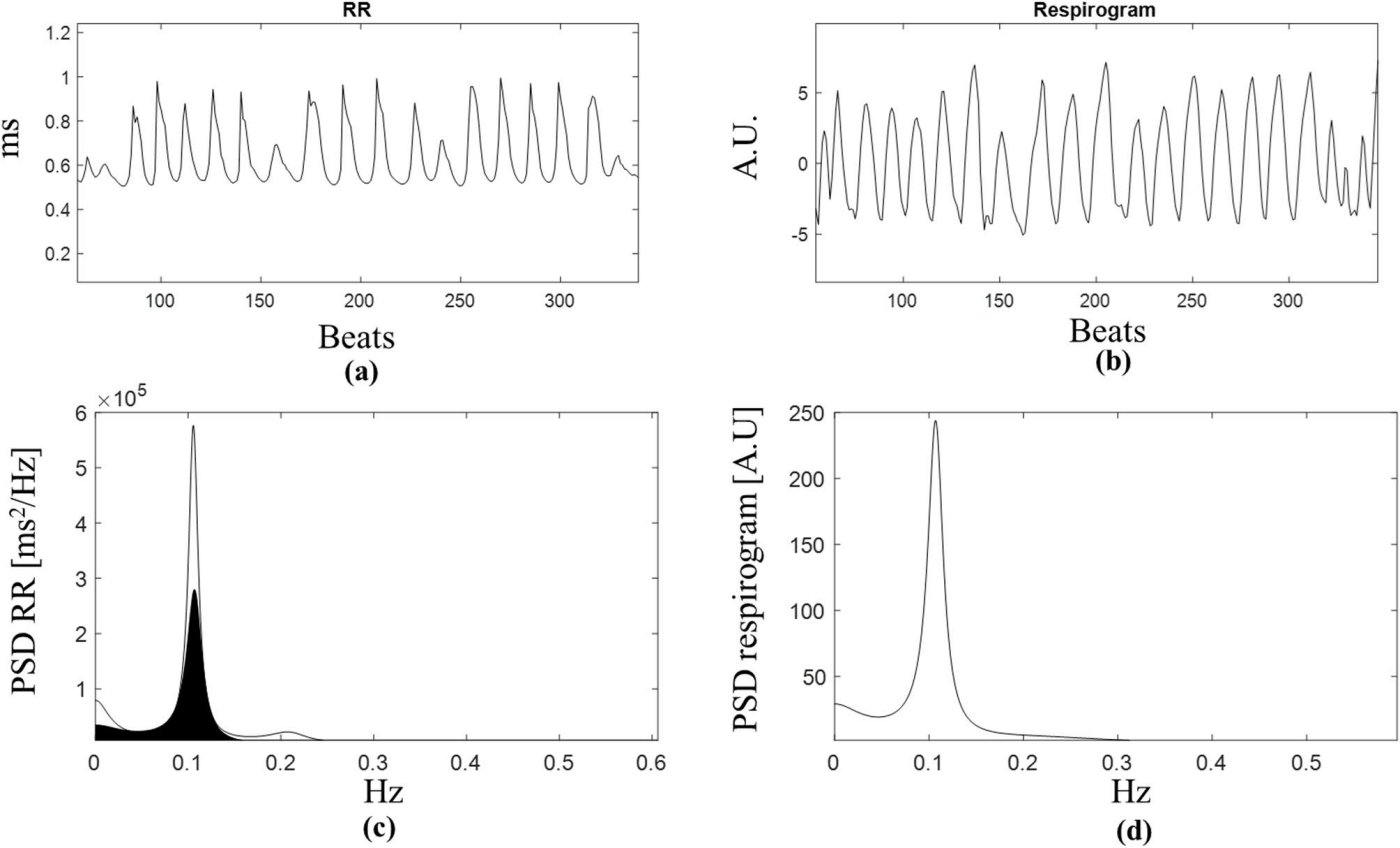
(**a**) The HRV signal at REST and (**b**) the associated respirogram signal for a participant. (**c**) The auto spectrum of the RR signal is represented with the portion of the spectrum that is due to the contribution of the respiration activity (black area) overlapped. (**d**) Auto spectrum of the respirogam.

Secondly, the bivariate-AR analysis was also applied to the same RR signal segments and the corresponding segments of the detrended respirogram. This approach is commonly used to highlight linear frequency relationships between the two considered signals and offers the possibility to disentangle the contribution of the respiratory activity in the total heart rate variability^20,44,45^. Specifically, the HRV signal can be modeled as the sum of two contributions: the RSA (deterministic), caused by the respiratory activity, which can be seen as a contribution in the HRV frequency content coherent with respiration, and the not-coherent component, an intrinsic stochastic activity of the system. The usefulness of this approach is shown in Fig. 2, where the contribution of the respiratory activity is completely overlapped with the traditional LF component of the HRV and may be erroneously attributed to non-vagal contributions.

The bivariate-AR was modeled for each signal segment using the formulation^38,46^ in Eq. 4

4 $$\:Y\left(n\right)=-{\sum\:}_{i=1}^{p}A\left(i\right)Y\left(n-i\right)+U\left(n\right)Y\left(n\right)=\left[\genfrac{}{}{0pt}{}{{y}_{1}\left(n\right)}{{y}_{2}\left(n\right)}\right],\:\:A\left(i\right)=\left[\genfrac{}{}{0pt}{}{{a}_{11}\left(i\right)}{{a}_{12}\left(i\right))}\genfrac{}{}{0pt}{}{{a}_{21}\left(i\right)}{{a}_{22}\left(i\right))}\right],\:\:U\left(n\right)=\left[\genfrac{}{}{0pt}{}{{u}_{1}\left(n\right)}{{u}_{2}\left(n\right)}\right]$$

In this case, the processed series are in the form of vector Y(n), the matrix A(i) of order p contains the model coefficients (2*2*p), and U(n) is the vector of the residual terms. The model was estimated using the Yule–Walker equations solved through the Levinson–Wiggins–Robinson algorithm, and the model order p was again estimated between 7 and 15 by applying the AIC criterion and verifying the whiteness of the residuals. Transforming the estimated model into the frequency domain, the PSD matrix can be obtained. The bivariate model disentangles the contribution of the respiratory activity ($\:{y}_{2}(n$) = respirogram) from the HRV series ($\:{y}_{1}\left(n\right)$= RR series). Using this approach, three parameters were extracted in each phase, specifically the power of the coherent component (PCOH), representing the RSA index^20^, and the power of the not-coherent component (PNCOH).

#### Nonlinear HRV parameters

Additionally, the following nonlinear analysis methods were also applied to the HRV signal in each protocol phase and previously defined time windows: Sample Entropy (SamEn), Detrended Fluctuation Analysis (DFA), and Poincarè plot (or recurrence plot)^14,47,48^. The nonlinear analysis was conducted using scripts adapted from open-source codes [https://github.com/jramshur/HRVAS]^49^ and custom implementations.

Entropy analysis quantifies the irregularity and complexity of the HRV signal and of its fluctuations. Specifically, the SamEn, which can be robustly estimated from short signals (at least 1 min of RR signal is required, according to^14^), was determined using an embedding dimension m = 2 and a tolerance threshold *r* = 0.2 of the standard deviation of the signal.

From the DFA, which characterizes the long-range fluctuation of the HRV signal, two slope parameters were extracted: a_1_ from 4 to 12 heartbeats and a_2_ from 12 to 64 heartbeats^13^. The second slope requires at least 2 min of RR intervals to be correctly estimated^14^.

From the Poincarè plot, comparing the variation between consecutive RR intervals, we extracted the standard deviation of the short-term RR series variability (SD1), the standard deviation of the long-term RR series variability (SD2), and their ratio SD1/SD2^48^.

#### Respiration parameters

Two parameters in the time domain were extracted from the series of respiratory period duration estimated as described in Sect. Signal pre-processing. Also in this case, a 4-minute segment centered in each protocol phase was used to compute the mean interval between consecutive breaths (meanBB) and their standard deviation (stdBB).

#### EDA parameters

Finally, from the EDA signal, processed through the Ledalab toolbox^22^, three phasic indexes of the EDA were computed considering a 4-minute window at the center of each protocol phase. Specifically, in each segment, the number of phasic peaks (nSCR), the mean amplitude of the phasic driver (SCR), and the sum of the phasic response amplitudes (AmpSum) were extracted. Parameters based on the tonic component were not considered in this analysis, since an influence of wearing time was observed for most participants (i.e., a constantly increasing tonic level).

### Statistical analysis

First, to analyze the modulation of physiological indexes due to different intensities of mental engaging and stress levels, we compared the entire sample’s (*N* = 60) parameter distributions among the four conditions (‘REST’, ‘MIST’, ‘MGT’, and ‘VS’) with the hypothesis that the MIST, MGT, and VS tasks elicit a physiological activation with respect to REST. The statistical analysis was carried out using the non-parametric Friedman’s test, since most parameters were not normally distributed, according to the Kolmogorov-Smirnov test and quartile-quartile plot exploration. A post-hoc analysis was also conducted to identify specific differences between conditions at a significance level of 0.05, after p-value correction using Bonferroni’s method. The same approach was repeated within each group (i.e., CONTROL, STRESS), in order to observe possible different patterns induced by the introduction of time and social pressure in the MIST task. To further investigate the role of such additional stressors in MIST and highlight possible differences between the two experimental groups, the variation with respect to the REST condition was computed (MIST–REST, MGT–REST, and VS–REST) for each parameter and compared between the two groups using a Mann-Whitney U-test within each task.

To strengthen the interpretation of the results, Cohen’s non-parametric effect size (r) was computed^50^. The suggested interpretation (> 0.1 small, > 0.3 medium, > 0.5 large) was adopted.

To explore the relationship between physiological parameters and perceived stress due to MIST manipulations, Spearman’s correlation (rho) was estimated between changes in physiological parameters (MIST–REST) and SUDS levels (post–pre MIST) measured before and during or after MIST. Furthermore, Spearman’s correlation was also estimated between the variation of physiological parameters during MIST and MGT with respect to REST and the corresponding variation of cortisol levels in terms of percentage variation from baseline computed as 100* $\:\frac{{t}_{0}\:-\:{t}_{\mathrm{1,2}}}{{t}_{0}}\:.$

Additionally, with the aim of understanding whether a combination of the analyzed parameters can provide a clear distinction between REST and the three cognitive tasks, we applied a multivariable analysis considering the parameters showing significant variations across tasks. Specifically, we first evaluated Pearson’s correlation between indices to exclude highly correlated pairs of features (|Pearson’s r| > 0.75) and normalized (z-score) the remaining ones. The features from all participants were used to train three binary Random Forest (RF) models with 100 estimators and leave-one-subject-out cross-validation. The procedure was repeated 100 times, using different random seeds. Models were trained to distinguish the following condition pairs: REST vs. MIST, REST vs. MGT, and REST vs. VS. We preferred this approach to a multinomial classification because our goal was not obtaining a model able to separate the specific experimental conditions we examined but, rather, to determine which set of features better allows to distinguish each kind of task from the resting state and explain possible reasons behind that. To evaluate model performance, classification accuracy was computed and averaged across procedure repetitions.

Since the aim of this analysis was to identify a set of features that, combined, may better characterize the observed physiological responses, we applied the Shapley (SHAP)^41^ approach to analyze feature importance for the three described models, thereby explaining the contribution of the most relevant features.

## Results

The assessment of the initial stress level as measured by the PSS, POMS, and SUDS showed homogeneous starting levels for the two groups. Specifically, the CONTROL group reported a mean response (between 1 and 5) to PSS of 3.11 (SD = 0.35) and a mean response (between 1 and 5) to POMS of 2.17 (SD = 0.44), while the STRESS group reported a mean PSS of 3.05 (SD = 0.33) and a mean POMS of 2.08 (SD = 0.46). As for the initial SUDS levels on a scale from 0 to 100, CONTROL group indicated a mean of 33.6 (SD = 20.5), the STRESS group indicated a mean level of 28.9 (SD = 19.1).

The non-parametric Wilcoxon signed rank test was applied to test whether the Cortisol concentrations collected at t1 and t2 varied significantly with respect to t0. Cortisol concentrations collected at t1 (ALL : median = 0.26 µg/dL, IQR = 0.20 µg/dL; CONTROLS: median = 0.27 µg/dL, IQR = 0.14 µg/dL; STRESS: median = 0.25 µg/dL, IQR = 0.22 µg/dL) and t2 (ALL : median = 0.25 µg/dL, IQR = 0.20 µg/dL; CONTROLS: median = 0.24 µg/dL, IQR = 0.16 µg/dL; STRESS: median = 0.28 µg/dL, IQR = 0.28 µg/dL) did not show any significant variation with respect to baseline values at t0 (ALL : median = 0.26 µg/dL, IQR = 0.16 µg/dL; CONTROLS: median = 0.26 µg/dL, IQR = 0.14 µg/dL; STRESS: median = 0.26 µg/dL, IQR = 0.21 µg/dL), neither considering the whole population (t1-t0: *p* = 0.349, *r* < 0.001; t2-t0: *p* = 0.123, *r* = 0.004), neither considering the STRESS (t1-t0: *p* = 0.202, *r* = 0.008; t2-t0: *p* = 0.147, *r* = 0.014) and the CONTROL group (t1-t0: *p* = 0.886, *r* = 0.014; t2-t0: *p* = 0.523, *r* = 0.006) separately.

### Heart rate variability parameters

Table 1 reports median, 25th, and 75th percentile values of all the HRV indexes, for the entire sample and the two groups separately. While a general decreasing trend from REST to all the other conditions is observable for the three time domain parameters, Friedman’s test identified significant differences only for meanRR and stdRR when the complete sample was considered (meanRR *p* < 0.001; stdRR *p* = 0.004; RMSSD *p* = 0.121). Concerning the mean RR interval duration, post-hoc analysis with Bonferroni’s correction showed a significantly decreased value from REST to MIST (*p* = 0.009↓, *r* = 0.468) and to MGT (*p* = 0.011↓, *r* = 0.473), and an increase from MIST to VS (*p* = 0.043↑, *r* = 0.435). A significant decrease with moderate effect size was also observed for the stdRR from REST to all the other protocol phases (Rest to MIST, *p* < 0.001↓, *r* = 0.498; Rest to MGT, *p* = 0.023↓, *r* = 0.455, and Rest to VS, *p* = 0.023↓, *r* = 0.415).

**Fig. 3:**
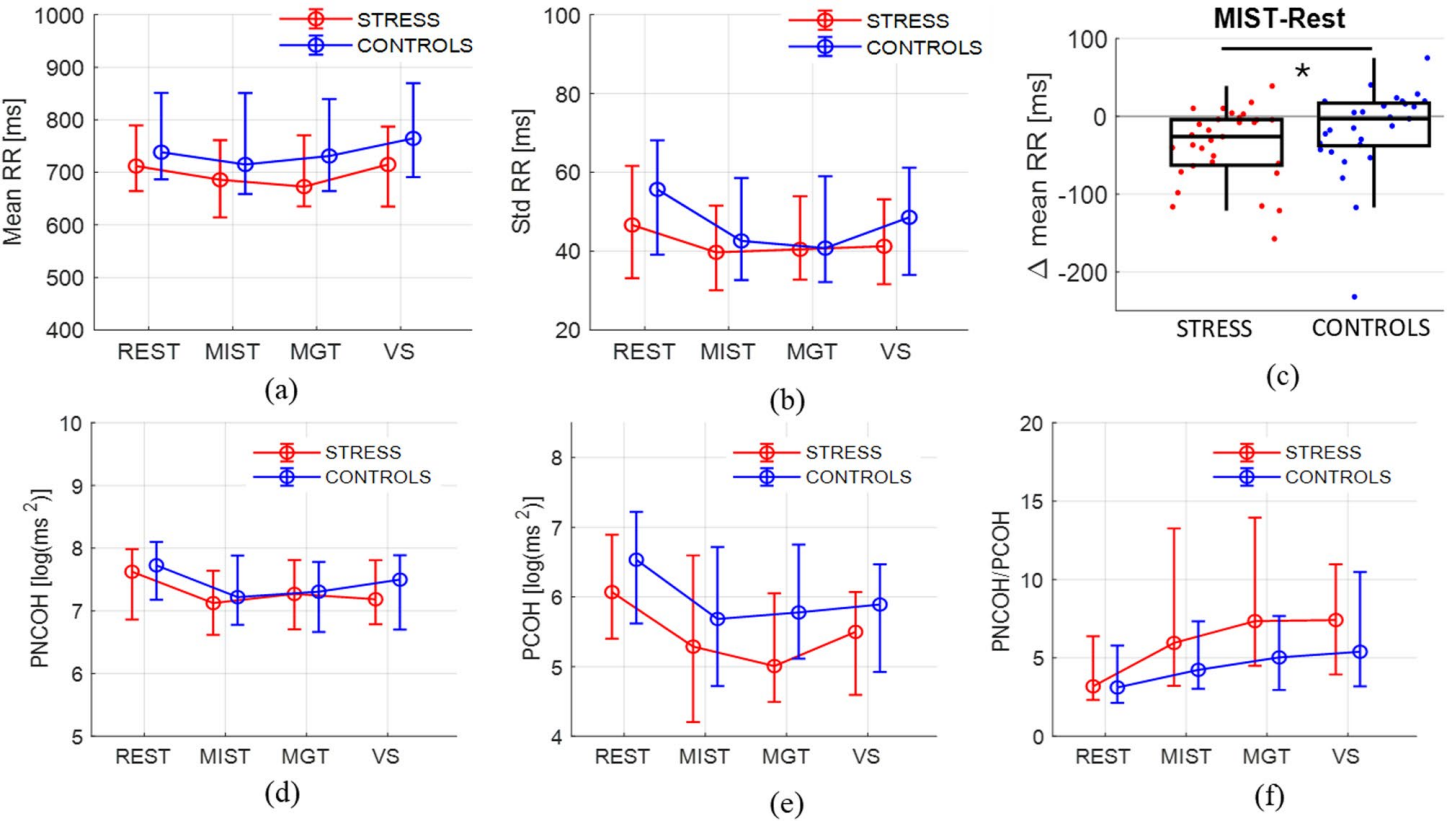
HRV parameters showing significant modulations (Friedman’s Test *p* < 0.05). Panels (**a**) and (**b**) show the median, 25 and 75th percentiles values of time domain parameters, respectively the mean RR and the std RR, for the two groups in each protocol phase. Panel (**c**) represents the variation of the mean RR during MIST with respect to Rest for STRESS in red and CONTROLS in blue. * Indicates a significant difference *p* = 0.03). Panels (**d**–**f**) display the median, 25 and 75th percentiles values of frequency domain parameters estimated using the bivariate approach, respectively the power not coherent with respiration, the RSA estimation and their ratio.

Interestingly, repeating the analysis for the two groups separately revealed different modulations shown in Fig. 3(a) and (b). Specifically, in the CONTROL group, Friedman’s test detected statistically significant differences for the meanRR (*p* = 0.033) and stdRR (*p* = 0.008) parameters, not for RMSSD (*p* = 0.121). In the STRESS group, only meanRR showed significant variations (*p* = 0.002). Pairwise corrected comparisons for the CONTROL group identified a significant increase in meanRR from MGT to VS phases (*p* = 0.036↑, *r* = 0.620) and significant decreases in stdRR from REST to both MIST (*p* = 0.007↓, *r* = 0.576) and MGT (*p* = 0.049↓, *r* = 0.560). In the STRESS group, instead, the mean RR interval significantly decreased from REST to MIST (*p* = 0.002↓, *r* = 0.672) and to MGT (*p* = 0.019↓, *r* = 0.616) phases. This different behavior is also confirmed by the significant difference (with small effect size) between the two groups in terms of ∆meanRR observed in the MIST task (MIST–REST, *p* = 0.03, *r* = 0.273), as depicted in Fig. 3(c).

The frequency-domain analysis based on the univariate AR model to estimate LF and HF powers and the corresponding normalized values showed a decreasing, yet not significant, trend for LF, HF and LF/HF, mostly due to a decrease in total power of the HRV signal during the three tasks with respect to REST, while we observed unchanged normalized powers.

**Table 1:** Linear HRV parameters in time and frequency domain (median and 25th-75th percentiles values).

		ALL				CONTROL				STRESS			
		REST	MIST	MGT	VS	REST	MIST	MGT	VS	REST	MIST	MGT	VS
meanRR (ms)	*med*	726.28	693.87*	696.44*	**724.49 #**	738.35	714.97	731.03	**764.62 §**	711.92	685.89*	672.83*	714.95
	*25th -75th*	673.11-807.61	631.08-803.87	645.76-805.84	652.74-817.28	686.55-851.06	658.78-850.87	664.29-839.33	690.88-869.64	664.28-789.21	614.31-761.16	635.24-770.59	634.92-786.91
StdRR (ms)	*med*	47.88	41.58*	40.62*	43.65*	55.67	42.60*	40.77*	48.60	46.64	39.71	40.46	41.24
	*25th -75th*	37.25–65.03	31.10-52.41	32.51–55.99	32.62–57.60	39.14–68.11	32.66–58.54	32.15–58.97	33.97–61.14	33.12–61.64	30.05–51.56	32.74–53.93	31.59–53.15
RMSSD (ms)	*med*	24.64	25.46	23.31	714.95	29.42	26.24	23.74	30.83	22.57	21.27	22.50	26.08
	*25th -75th*	17.78–41.45	14.11–40.94	16.07–39.52	634.92-786.91	19.81–43.84	17.30-39.55	16.08–41.07	20.33–43.01	15.41–32.84	12.97–40.86	14.49–37.30	15.44–34.39
HF (ms^2)	*med*	260.87	247.51	198.54	238.34	272.76	265.23	196.47	258.45	172.07	157.56	200.60	233.11
	*25th -75th*	106.31-546.71	89.32-451.22	89.23-449.53	99.00-467.03	201.71-812.33	102.78-580.24	104.47-505.33	99.62-589.72	62.86-501.81	84.40-384.41	55.71-412.07	68.35-367.86
LF (ms^2)	*med*	680.32	626.87	589.70	616.36	1006.85	716.00	664.31	695.08	568.09	615.88	453.40	532.34
	*25th -75th*	425.49-1488.29	308.76-1121.90	260.58-1123.50	285.18-1288.90	513.39-1736.09	357.66-1319.16	281.97-1251.80	316.26-1682.92	259.51-1204.11	263.86-1086.49	250.30-911.81	237.35-1053.43
LF/HF	*med*	3.23	3.08	3.63	2.28	2.85	2.85	3.25	1.75	3.64	3.39	3.91	2.69
	*25th -75th*	1.70–6.28	1.63–5.60	1.24–6.42	1.43–6.32	1.59–5.79	1.88–4.76	1.31–4.91	1.34–7.04	1.97–7.66	1.54–5.79	0.98–7.51	1.43–5.31
HF norm (%)	*med*	23.80	24.54	21.61	30.53	25.98	25.96	23.55	36.32	21.53	22.76	20.38	27.09
	*25th -75th*	13.75–37.03	15.16-38.00	13.47–44.61	13.68–41.19	14.76–38.73	17.45–34.79	16.94–43.25	12.56–43.07	11.69–33.64	14.72–39.45	11.75–50.53	15.91–41.14
LF norm (%)	*med*	76.20	75.46	78.39	69.47	74.02	74.04	76.45	63.68	78.47	77.24	79.62	72.91
	*25th -75th*	62.97–86.25	62.00-84.84	55.39–86.53	58.81–86.32	61.27–85.24	65.21–82.55	56.75–83.06	56.93–87.44	66.36–88.31	60.55–85.28	49.47–88.25	58.86–84.09
PCOH (RSA index) (ms^2)	*med*	522.38	276.24*	250.26*	272.55*	688.05	294.07*	322.85*	361.91*	432.51	197.82*	149.56*	244.25*
	*25th -75th*	252.37-1194.76	80.53–778.80	121.80-482.76	100.91-531.01	276.11-1382.40	112.23-826.34	166.35-855.83	141.31-645.02	222.22-983.63	66.87-734.05	89.90-425.82	98.95-433.86
PNCOH (ms^2)	*med*	2110.22	1317.51*	1468.02*	1495.98*	2268.16	1365.16	1489.07	1805.94*	2047.04	1242.03*	1436.54	1319.50*
	*25th -75th*	1188.16-3102.47	758.11-2287.3	781.79-2442.44	830.12-2519.71	1310.44-3292.11	878.20-2647.92	782.46-2397.36	812.16-2657.25	955.16-2939.50	747.41-2078.12	815.66-2464.96	885.07-2460.71
PNCOH/PCOH	*med*	3.15	4.99*	5.76*	6.52*	3.12	4.24	5.03	5.39*	3.19	5.95*	7.35*	7.42*
	*25th -75th*	2.20–5.96	3.18–11.23	3.43–11.58	3.50-10.74	2.13–5.79	3.03–7.33	2.95–7.67	3.18–10.47	2.31–6.37	3.21–13.25	4.49–13.94	3.94–10.97

Symbols identify statistical differences: * different from REST; # different from MIST; § different from MGT.

Considering the bivariate analysis for the whole sample, PNCOH and PCOH showed a decreasing trend going from REST to task conditions, while the ratio PNCOH/PCOH increased. These trends were found significant according to the Friedman’s test (PNCOH *p* < 0.001; PCOH *p* < 0.001; PNCOH/PCOH *p* < 0.001) and the post-hoc Bonferroni-corrected analysis was performed. Specifically, significant decreases in PNCOH were observed between REST and all the tasks with mainly moderate effect size (Rest to MIST, *p* < 0.001↓, *r* = 0.540; Rest to MGT, *p* = 0.018↓, *r* = 0.359, Rest to VS, *p* < 0.001↓, *r* = 0.453), while no differences were found among the three tasks. Similar results were obtained for PCOH (RSA index) with larger effect size (Rest to MIST, *p* < 0.001↓, *r* = 0.590; Rest to MGT, *p* < 0.001↓, *r* = 0.682, Rest to VS, *p* < 0.001↓, *r* = 0.668), while the ratio significantly increased in the same tasks reporting from moderate to large effect sizes (Rest to MIST, *p* = 0.035↑, *r* = 0.392; Rest to MGT, *p* < 0.001↑, *r* = 0.536; Rest to VS, *p* < 0.001↑, *r* = 0.562).

These results are confirmed at the single-group level. For the CONTROL group, the Friedman’s test highlighted the presence of significant variations among tasks in PNCOH (*p* = 0.012), PCOH (*p* < 0.001), and PNCOH/PCOH (*p* = 0.043). In particular, only the PNCOH decrease during VS with moderate effect size survived to Bonferroni’s correction (Rest to VS, *p* = 0.0137↓, *r* = 0.488), the PCOH significantly decreased in all the tasks with respect to REST with large effect size (Rest to MIST, *p* = 0.0033↓, *r* = 0.612; Rest to MGT, *p* = 0.0007↓, *r* = 0.733, Rest to VS, *p* = 0.0001↓, *r* = 0.636), while their ratio significantly increased only in VS with respect to REST (*p* = 0.0264↑, *r* = 0.584). The same differences were more marked in the STRESS group, particularly for the PINCOH/PCOH parameter (Friedman’s test: PNCOH *p* = 0.010; PCOH *p* < 0.001; PNCOH/PCOH *p* < 0.001). More in detail, after p-values corrections, significant decreases in PNCOH were observed during MIST (*p* = 0.014↓, *r* = 0.524) and VS with respect to REST (*p* = 0.035↓, *r* = 0.402) and for PCOH, again, from REST to MIST (*p* = 0.002↓, *r* = 0.591), MGT (*p* = 0.002↓, *r* = 0.679), and VS (*p* = 0.002↓, *r* = 0.707), while no differences were found among cognitive tasks. The STRESS group also showed an increase in PNCOH/PCOH for all the tasks with respect to REST (Rest to MIST, *p* = 0.047↑, *r* = 0.461; Rest to MGT, *p* < 0.001↑, *r* = 0.598; Rest to VS, *p* = 0.007↑, *r* = 0.535). The distribution of these parameters are shown in Fig. 3 (d), (e) and (f).

In the frequency domain, there were no significant differences among the groups.

Table 2 reports median, 25th, and 75th percentile values for the nonlinear HRV indexes for the whole population and the two groups separately. SamEn was found to be significantly affected by the protocol phase for the entire sample and for each group (All: *p* < 0.0001, CONTROL: *p* < 0.001, STRESS: *p* < 0.001). On the entire sample, after p-values correction, we found a significant increase in SamEn during all tasks with respect to REST conditions with an effect size from moderate to large (Rest to MIST, *p* < 0.001↑, *r* = 0.539; Rest to MGT, *p* = 0.022↑, *r* = 0.359; Rest to VS, *p* < 0.001↑, *r* = 0.710) and during VS compared to MGT (*p* = 0.028↑). Analyzing the groups separately, we notice a significant increase in SamEn in both cases, from REST to MIST and from REST to VS (CONTROL: Rest to MIST, *p* = 0.013↑, *r* = 0.572, Rest to VS, *p* < 0.001↑, *r* = 0.661; STRESS: Rest to MIST, *p* = 0.009↑, *r* = 0.514; REST to VS, *p* < 0.001↑, *r* = 0.750). No differences were observed between the two groups.

**Table 2:** Nonlinear HRV parameters in time and frequency domain (median and 25th-75th percentile values).

		ALL				CONTROL				STRESS			
		REST	MIST	MGT	VS	REST	MIST	MGT	VS	REST	MIST	MGT	VS
SD1	*med*	0.017	0.018	0.017	0.020	0.021	0.019	0.017	0.022	0.016	0.015	0.016	0.018
	*25th -75th*	0.013–0.029	0.010–0.029	0.011–0.028	0.012–0.027	0.014–0.031	0.012–0.028	0.011–0.029	0.014–0.030	0.011–0.023	0.009–0.029	0.010–0.026	0.011–0.024
SD2	*med*	0.066	0.056*	0.055*	0.057*	0.075	0.058*	0.056	0.067	0.064	0.053*	0.054	0.056
	*25th -75th*	0.051–0.086	0.043–0.071	0.044–0.072	0.045–0.077	0.054–0.091	0.044–0.072	0.044–0.076	0.046–0.082	0.046–0.083	0.040–0.068	0.045–0.071	0.042–0.071
SD1/SD2	*med*	0.280	0.314*	0.291	0.339*	0.292	0.336	0.292	0.398*	0.253	0.294	0.289	0.306*
	*25th -75th*	0.239–0.341	0.253–0.396	0.236–0.403	0.265–0.423	0.249–0.351	0.274–0.404	0.246–0.366	0.266–0.432	0.229–0.307	0.220–0.391	0.225–0.430	0.262–0.362
SamEn	*med*	1.239	1.409*	1.325*	**1.504** *§	1.286	1.407*	1.402	1.542*	1.208	1.411*	1.226	1.487*
	*25th -75th*	1.030–1.398	1.239–1.649	1.116–1.528	1.257–1.694	1.142–1.525	1.316–1.658	1.165–1.525	1.303–1.781	1.013–1.347	1.045–1.646	0.998–1.534	1.144–1.569
a_1_	*med*	1.392	1.295	1.340	1.222	1.385	1.276	1.330	1.190	1.457	1.407	1.351	1.320
	*25th -75th*	1.186–1.505	1.112–1.495	1.091–1.526	1.079–1.442	1.133-1.500	1.156–1.481	1.091–1.522	1.074–1.350	1.239–1.518	1.094–1.538	1.072–1.528	1.092–1.443
a_2_	*med*	0.808	0.770	0.837	0.802	0.787	0.747	0.801	0.800	0.831	0.776	0.870	0.828
	*25th -75th*	0.686–0.929	0.634–0.908	0.734–0.936	0.645–0.983	0.661–0.892	0.638–0.899	0.737–0.949	0.677–0.955	0.722–1.011	0.619–0.908	0.714–0.930	0.637–0.988

Symbols identify statistical differences: * different from REST; # different from MIST; § different from MGT.

The extracted DFA indices did not show any significant differences. As for the recurrence plot analysis, the SD1 was not affected by the protocol phases for both the whole sample and the group-wise analysis. SD2 showed a general decrease from REST to all the other protocol phases, while the SD1/SD2 ratio increased. Specifically, considering the complete sample (Friedman’s test *p* < 0.001), the SD2 decrease was significant for each protocol phase with respect to REST (Rest to MIST, *p* < 0.001↓, *r* = 0.539; Rest to MGT, *p* = 0.04↓, *r* = 0.460; Rest to VS, *p* = 0.007↓, *r* = 0.451), while in the CONTROL group, after correction for multiple comparisons, the difference was significant only between REST and MIST (*p* = 0.013↓, *r* = 0.604) as for the STRESS group (*p* = 0.026, *r* = 0.465). The ratio SD1/SD2, instead, increased during the tasks with respect to REST (Friedman test: ALL: *p* < 0.001, CONTROL: *p* = 0.035, STRESS: *p* = 0.006). Specifically, for the entire sample, the increase was significant for both MIST (*p* = 0.011↑, *r* = 0.457) and VS with respect to REST (*p* < 0.001↑, *r* = 0.537), whereas separating the two groups, only VS showed a higher value than the REST condition for both groups (CONTROL: *p* = 0.02, *r* = 0.500, STRESS: *p* = 0.003, *r* = 0.598).

### Respiration parameters

From the thoracic belt signal, we estimated the mean interval between consecutive breaths (meanBB) and their variability (stdBB) for each protocol phase, reported in Table 3. Considering the overall sample, Friedman’s test identified statistically significant differences due to task effect for both the indexes (meanBB *p* < 0.001, stdBB *p* < 0.001). Specifically, both the parameters significantly decreased from REST to MIST, MGT, and VS (meanBB: Rest to MIST, *p* < 0.001↓, *r* = 0.703; Rest to MGT, *p* < 0.001↓, *r* = 0.688, Rest to VS, *p* < 0.001↓, *r* = 0.750; stdBB: Rest to MIST, *p* = 0.001↓, *r* = 0.540; Rest to MGT, *p* = 0.002↓, *r* = 0.538, Rest to VS, *p* < 0.001↓, *r* = 0.643), while no differences were observed among the three tasks after Bonferroni’s correction.

Similar patterns were found in the two groups, where Friedman’s test reported significant variations (CONTROL: meanBB *p* < 0.001, stdBB *p* < 0.001; STRESS: meanBB *p* < 0.001, stdBB *p* < 0.001), which were confirmed by the post-hoc analysis with correction for multiple comparisons. The meanBB was significantly decreased during MIST, MGT, and VS with respect to REST for both the CONTROL (Rest to MIST, *p* < 0.001↓, *r* = 0.713; Rest to MGT, *p* = 0.003↓, *r* = 0.761; Rest to VS, *p* < 0.001↓, *r* = 0.833) and the STRESS group (Rest to MIST, *p* < 0.001↓, *r* = 0.700; Rest to MGT, *p* = 0.035↓, *r* = 0.613; Rest to VS, *p* < 0.001↓, *r* = 0.676). As for the variability of the respiratory period, a significant decrease was observed from REST to MIST (*p* = 0.036↓, *r* = 0.516) and to VS (*p* < 0.001↓, *r* = 0.677) in the CONTROL group, while the decrease in the STRESS group was significant from REST to MGT (*p* = 0.01↓, *r* = 0.535) and to VS (*p* < 0.001↓, *r* = 0.623) only. For the respiratory parameters, no statistical differences were observed between experimental groups.

### EDA parameters

**Fig. 4:**
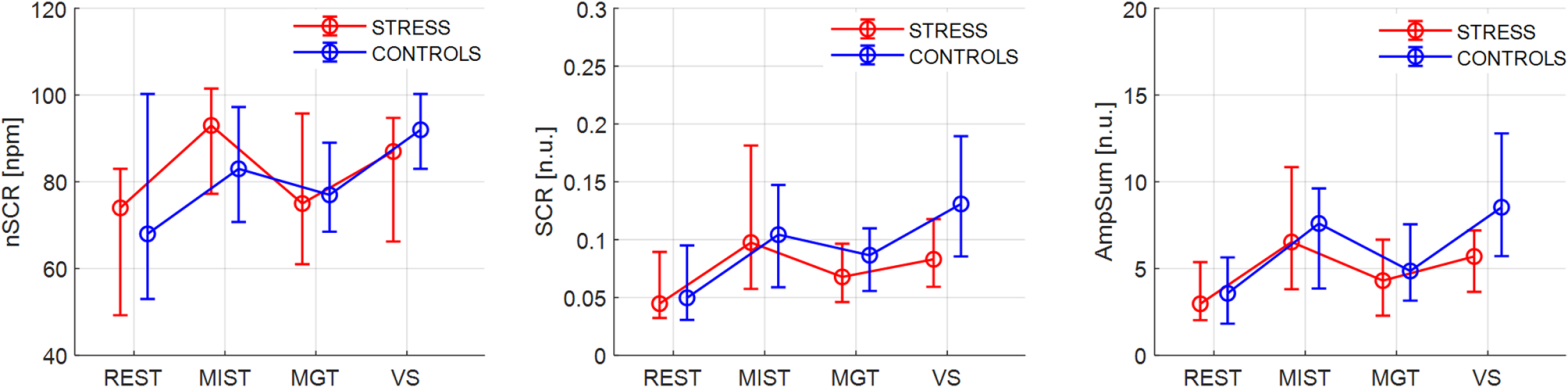
Median, 25th and 75th percentiles values for the EDA derived parameters in each protocol phase for the STRESS and the CONTROL group. The nSCR is reported in number per minute (npm), the SCR and the AmpSum are represented in normalized units (N.U.).

The activation parameters of interest extracted from the phasic component of the EDA signal are reported in Table 3 as median values and 25th -75th percentiles and represented in Fig. 4. Concerning the whole sample, Friedman’s test revealed a significant effect of the protocol phases for all three parameters (nSCR *p* < 0.001; SCR *p* < 0.001 and AmpSum *p* < 0.001). Post-hoc Bonferroni-corrected analysis showed a significant difference between each of the three tasks and the REST condition. Specifically, a significant increase in nSCR was observed during MIST (*p* < 0.001↑, *r* = 0.566), MGT (*p* = 0.042↑, *r* = 0.393), and VS (*p* < 0.001↑, *r* = 0.589). As for SCR, significant increases were observed during all the tasks with respect to REST with mostly large effect size (REST to MIST, *p* < 0.001↑, *r* = 0.802; REST to MGT, *p* = 0.002↑, *r* = 0.527; and REST to VS, *p* < 0.001↑, *r* = 0.693) and in VS with respect to MGT (MGT to VS, *p* = 0.001↑, *r* = 0.562), suggesting decreased activation during MGT, even if non-significant when compared to MIST. Similar results were obtained analyzing the AmpSum index (REST to MIST, *p* < 0.001↑, *r* = 0.802; REST to VS, *p* < 0.001↑, *r* = 0.698; REST to MGT, *p* = 0.005↑, *r* = 0.540; MGT to VS, *p* < 0.001↑, *r* = 0.550).

**Table 3:** Respiration variability metrics and EDA derived phasic parameters (median and 25–75th percentiles values).

		All				Control				Stress			
Respiration		REST	MIST	MGT	VS	REST	MIST	MGT	VS	REST	MIST	MGT	VS
meanBB (s)	*med*	3.88	3.22*	3.46*	3.14*	3.67	3.19*	3.33*	3.04*	3.99	3.33*	3.50*	3.20*
	*25th -75th*	3.29–4.59	2.85–3.66	3.00-3.90	2.83–3.57	3.35–4.58	2.83–4.03	2.94–3.91	2.83–3.59	3.02–4.57	2.87–3.62	3.11–3.86	2.83–3.56
stdBB (s)	*med*	0.99	0.75*	0.74*	0.54*	0.99	0.67*	0.77	0.62*	1.00	0.80	0.72*	0.52*
	*25th -75th*	0.71–1.60	0.43–1.03	0.48–1.13	0.37–0.94	0.72–1.70	0.42–1.05	0.50–1.09	0.37–0.84	0.70–1.47	0.44-1.00	0.48–1.15	0.39–1.03
EDA													
nSCR	*med*	70	88*	76*	87*	68	83	77	92*	74	93*	75	87*
	*25th -75th*	53–86	73.5–98.5	66–92	75.5–98	53-100.25	70.75–97.25	68.5–89	83-100.25	49.25-83	77.25–101.5	61-95.75	66.25–94.75
SCR	*med*	0.05	0.10*	0.07*	0.10*§	0.05	0.10*	0.09*	**0.13** *#§	0.04	0.10*	0.07	0.08*
	*25th -75th*	0.03–0.09	0.06–0.16	0.05–0.11	0.07–0.16	0.03–0.09	0.06–0.15	0.06–0.11	0.09–0.19	0.03–0.09	0.06–0.18	0.05–0.10	0.06–0.12
Amp Sum	*med*	3.19	6.97*	4.55*	6.40*§	3.58	7.60*	4.86	**8.54** *#§	2.97	6.53*	4.31	5.70*
	*25th -75th*	1.93–5.45	3.83–10.27	2.96–7.31	4.07–10.57	1.83–5.64	3.85–9.62	3.16–7.55	5.72–12.79	2.02–5.37	3.81–10.85	2.29–6.67	3.66–7.20

Symbols identify statistical differences: * different from REST; # different from MIST; § different from MGT.

Regarding the single-group level analysis, the CONTROL group showed significant variations in all the analyzed parameters (nSCR *p* = 0.001; SCR *p* < 0.001 and AmpSum *p* < 0.001). Interestingly, the number of SCR peaks increased significantly only during the VS task compared to the REST condition (*p* < 0.001↑, *r* = 0.655). Anyway, the mean SCR and AmpSum significantly increased from REST to MIST (SCR *p* = 0.001↑, *r* = 0.833; AmpSum *p* = 0.002↑, *r* = 0.829), from REST to VS (both with *p* < 0.001, *r* = 0.829 and *r* = 0.813), but also from MIST to VS (SCR *p* = 0.036↑, *r* = 0.432, AmpSum *p* = 0.049↑, *r* = 0.431) and from MGT to VS (both with *p* < 0.001, *r* = 0.737 and *r* = 0.729). Of note, the SCR also increases in MGT with respect to the REST phase (*p* = 0.036↑, *r* = 0.544).

The STRESS group showed a similar pattern, but with a more pronounced response to the MIST task, as expected. Friedman’s test again detected significant trends due to the different protocol phases (nSCR *p* = 0.017; SCR *p* < 0.001 and AmpSum *p* < 0.001). A significant increase in terms of nSCR was observed only from REST to MIST (nSCR *p* = 0.001↑, *r* = 0.690) and to VS (*p* = 0.025↑, *r* = 0.495). Similarly, significantly increased SCR and AmpSum were observed from REST to MIST (*p* < 0.001↑, *r* = 0.771 both indices) and from REST to VS (*p* < 0.001↑, *r* = 0.532 and *r* = 0.556 respectively). No differences were reported among the three cognitive tasks.

Comparing parameter changes with respect to the REST condition between groups, a significant difference with moderate effect size was found for both SCR (*p* = 0.006, *r* = 0.353) and AmpSum (*p* = 0.008, *r* = 0.340) indexes during the VS tasks. Specifically, a more pronounced increase in activation was observed in the CONTROL group.

### Correlation analysis

Correlations were investigated among physiological parameters that were significantly modulated by the protocol phases and the perceived stress level (SUDS scale). Changes in SUDS, from before to after the MIST task, were found negatively correlated with the modulation of the mean RR interval duration, both across the whole sample ( *p* = 0.005, Spearman’s rho=-0.355) and in the STRESS group (*p* = 0.009, Spearman’s rho=-0.464), but not in the control group. This suggests an increase in heart rate (i.e., decrease in mean RR interval) associated with an increased perceived stress. All the other parameters were not significantly correlated with SUDS changes.

As for the cortisol level, one subject was removed because it resulted in an outlier (larger than mean + 3*std). However, no significant correlation was found between any of the physiological parameters and cortisol level variation in t_1_ and t_2_ with respect to t_0_.

### Multivariable analysis

**Fig. 5:**
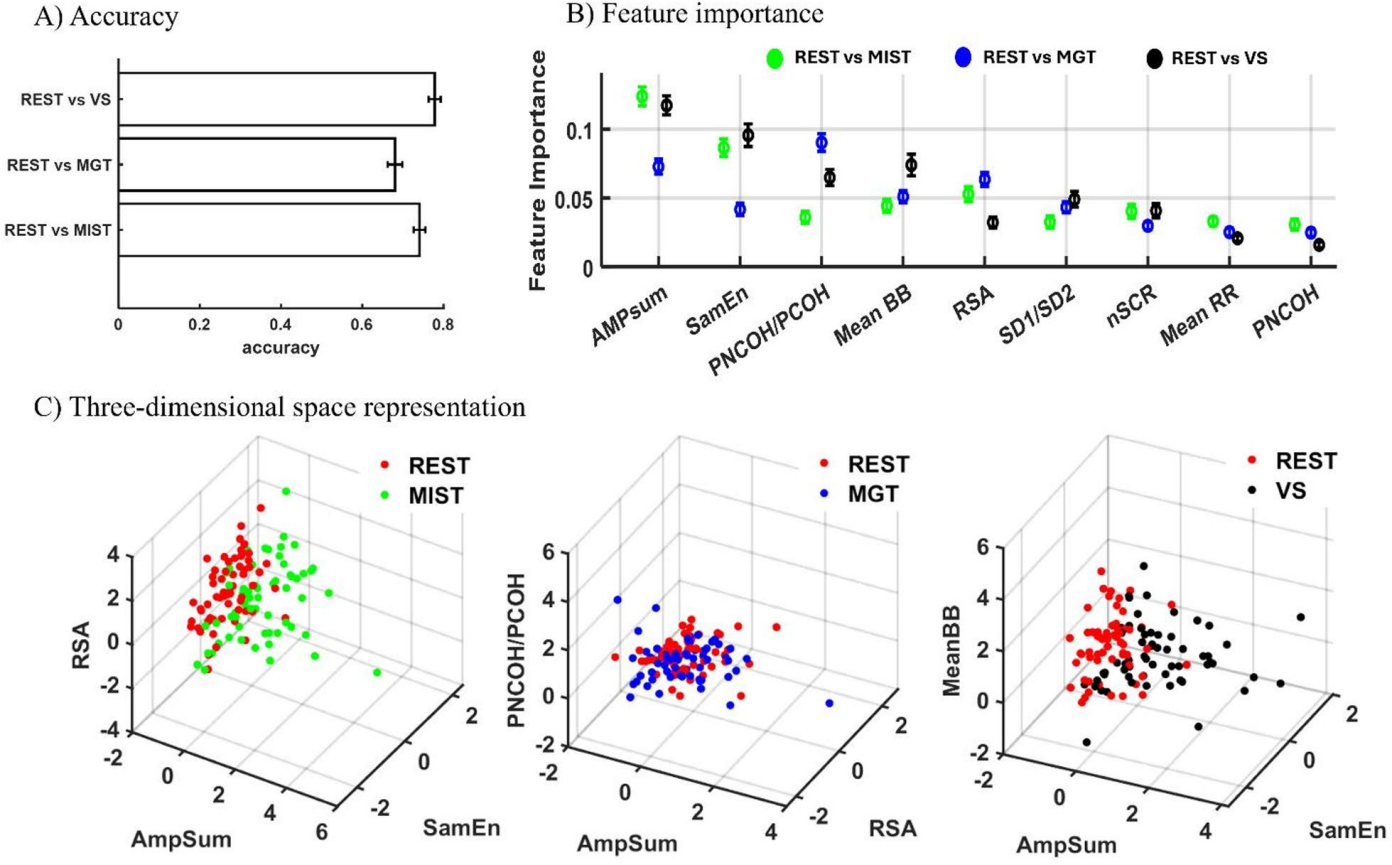
Multivariable analysis results. (**A**) Mean and standard deviation of accuracy values for the three binary models. (**B**) Mean and standard deviation of feature importance obtained from SHAP analysis, represented in order of descending importance considering the mean of the three tasks. (**C**) Scatter plots showing data distribution for each pair of conditions (REST vs. MIST, REST vs. MGT, and REST vs. VS) in the three-dimensional space represented by the three most important features for each model.

Figure 5 summarizes the main results for the multivariable analysis. Nine features were considered: nSCR, AmpSum, MeanRR, MeanBB, RSA index, PNCOH/PCOH, SamEn, and SD1/SD2. Figure 5. A shows the classification cross-validation accuracy of each binary model. In line with the univariate results, both the MIST and the VS task obtained moderate separation accuracy (MIST: 0.75 ± 0.014, VS: 0.78 ± 0.015), while the MGT was more difficult to separate from the REST condition, with an average accuracy of 0.68 ± 0.018. Since moderate differences emerged in the behaviors observed in the two groups, these results were obtained by training the models on the whole sample to maximize the number of available observations.

Figure 5B shows the results of Shapley analysis, pointing out the averaged importance of individual features (absolute Shapley values were considered for comparison) in each model. It is interesting to note that, depending on the task to be distinguished from REST, the contribution of each physiological parameter may change. Specifically, the EDA phasic parameter AmpSum highly contributes to distinguishing each task from REST.

From the HRV domain, the RSA index contributed to both MIST and MGT classification against REST, while SamEn was crucial for both MIST and VS. In the case of MGT, the PNCOH/PCOH was indicated as the most important feature. Finally, the mean distance between consecutive breaths (meanBB) contributed to VS classification. Figure 5C displays three-dimensional scatterplots built using the three most important features for each mental task as indicated by the SHAP evaluation.

## Discussion

In the current study, we investigated the ANS responses in a sample of healthy adults during an acute mental stress and cognitive stimulation protocol, analyzing a set of physiological parameters comprising cardiovascular, electrodermal, and respiratory activity. While the effect of acute mental stress and cognitive tasks has been largely investigated in recent literature^2,8,11,13,14^, results often appear conflicting depending on different factors, such as the study population, experimental protocol, and parameter estimation approach. Moreover, most of the previous literature has focused on HRV parameters, sometimes including EDA analysis, while respiratory activity is often overlooked. A second aim of the study was to understand if a different level of initially induced acute stress could affect the ANS responses throughout the experimental protocol. Therefore, the sample was randomly divided into two groups, one undergoing the original MIST and the other performing the same task in a less challenging modality. We also explored possible relationships between physiological parameters and psychometric as well as biochemical results. Finally, to identify a possible combination of parameters that could better characterize the response to each protocol condition, an RF-based multivariable analysis was also performed.

Our results confirmed that most of the physiological parameters included in the analysis were modulated by cognitive tasks, and depending on their characteristics and origins, their modulations possibly reflected different ANS response components. Regarding the HRV, in the whole group a decrease of the mean and the standard deviation of the RR intervals supported the hypothesis of a shift towards a sympathetic prevalence induced by mental stress, as previously reported^8,9^. It is worth noting that, considering the two groups separately, the meanRR was found significantly reduced during MIST only for the STRESS group, while in the CONTROL group, even if a decrease was observed, this was not significant, probably because of a larger variability. On the contrary, the stdRR significantly decreased only for the CONTROL group, but the decrease was not significant in the experimental group. Nevertheless, the two parameters, even if the significance resulted ‘group specific’, showed expected modulations, in line with the literature and the study hypothesis.

Interestingly, the RMSSD parameter, which has been described as one of the most sensitive indicators of acute stress^5^, did not show any significant modulation in our experiment. Similarly, frequency-domain HRV features were not considerably affected by the proposed protocol when estimated without considering the contribution of respiration, as also reported in the literature^20,47^. When disentangling the contribution of respiratory activity from the HRV signal, the task effect was significant, with a decreased RSA index suggesting withdrawal or deactivation of the parasympathetic (vagal) nervous system (PNS), in preparation for a demanding situation^20^. Nevertheless, this result should be interpreted also considering the observed changes in the respiration period (meanBB), which also modulate HRV parameters. In fact, the RSA index, when directly derived from the HF of the HRV spectrum, may be misinterpreted as a direct measure of vagal tone^51^. In our case, the use of the bivariate AR analysis mitigates this caveat by identifying the contribution of the respiration to the HRV spectrum at frequencies outside the expected HF range^20^. These results underscore the importance of integrating respiratory activity in frequency-domain HRV analysis, since breathing modulates HR and HRV^19^. Besides the frequency domain approaches, to learn more about RSA possible interpretation, new advanced tools have been developed able to estimate RSA and its dynamic^52^.

Linear HRV parameters were found sensitive to mental activities compared to the REST condition, but their changes were similar across the three tasks (MIST, MGT, and VS). Concerning the analysis of the HRV, it is worth noting that we used a 4-minute segment of RR time series to estimate both linear and nonlinear parameters. Although the reliability of estimates using at least 3 min of signals has been demonstrated in the literature^14^, we acknowledge that results related to LF power should be carefully considered since only a few cycles of the lowest LF frequency were captured.

Nonlinear HRV parameters, in particular SD2, SD1/SD2, and sample entropy, were also found sensitive to task manipulation. However, the interpretation of these parameters is not straightforward, and contrasting results have been reported in the literature. For example, while Pereira^13^ and Castaldo^14^ found a significant SD1 increase, other works reported decreases in short-term variability related to mental stress^47^, which resembles our results more closely, although such a decrease was not significant in our case (Friedman’s *p* = 0.121). As for the SD2 parameter, the literature and our results consistently suggest a significant decrease in long-range variability under stress and mental load^47^ .

The SD1/SD2 ratio, which significantly increased primarily during VS tasks in our case, has rarely been investigated in relation to mental stress. However, an increase in its value has been associated with the activation of the sympathetic nervous system, which is consistent with our hypothesis and results^48,53^. As for the SamEn index, in line with the results reported by Brugnera^8^ and Hao^47^, a significant increase in HRV complexity and irregularity under stress was identified. Although this index is widely used in literature, its interpretation in relation to ANS activity is still debated. To shed light on this, Lewis and Short^54^ measured the SamEn of the RR series during different levels of physical exercise. They reported an increase in signal complexity during exercise and a reduction during recovery. The authors concluded that changes in SamEn could be related to alterations in ANS control. In agreement with this interpretation, we speculate that an increase in SamEn, and therefore in signal irregularity, may also reflect a change in ANS control in the present study. Interestingly, the nonlinear parameters were differently modulated by the three tasks; specifically, a stronger change was observed during the MIST and VS phases compared to the REST, whereas the MGT was characterized by a lower variation. This pattern became particularly evident when analyzing the STRESS and CONTROL groups separately.

Concerning the analysis of EDA, we focused on its phasic component, as less affected by possible environmental factors and sensor wearing time. The three phasic indices were highly coherent and very sensitive to the different tasks; specifically, a strong sympathetic activation was evident during MIST and VS, as suggested by an increase in all the EDA parameters. Conversely, a return to almost initial levels was observed during MGT. In general, these results confirmed the close relationship between EDA and cognitive load^23,55^.

Additionally, a multivariable, RF-based analysis, followed by a feature importance evaluation, was performed on the entire sample to explore which combination of the considered parameters best characterizes the physiological response to each task relative to the resting condition. While the separation accuracy was only moderate in every case, ranging from an average of 0.68 for MGT to 0.78 for VS, some features clearly emerged as the most useful for classification. Specifically, AmpSum, RSA index, and SamEn were fundamental for separating the MIST condition from REST; AmpSum, PNCOH/PCOH, and RSA index were the most important features in the case of MGT; finally, AmpSum, SamEn, and MeanBB mostly contributed to the VS classification. Overall, the results of our multivariable analysis corroborate the physiological characterization of the responses to the proposed tasks provided by the univariable non-parametric statistics discussed above. In particular, they emphasize the importance of specific parameter combinations that should be prioritized when evaluating acute stress or mental activity, which may vary depending on the specific task of interest. Moreover, they suggest that a multimodal approach can provide a more complete view of the physiological mechanisms underlying stress and attention, given that most of those combinations were found to include features extracted from different signals.

When the two groups were analyzed separately, similar modulations of the ANS parameters were found. In fact, only a few parameters exhibited significant differences between groups, specifically the mean RR interval and the EDA phasic parameters. Even so, we were able to confirm the different effects of the two MIST implementations based on the stronger mean RR decrease (heart rate increase) for the STRESS group during MIST and the stronger relationship between this parameter and the variation in the perceived stress (SUDS score), which was not significant for the CONTROL group. Interestingly, also the phasic EDA activity was differently modulated in the two groups. In fact, a stronger, but non-significant activation during MIST was observed in the STRESS group, while, surprisingly, the CONTROL group showed a significantly higher activation during the attention test (VS task), while the experimental group was less activated. This result may suggest a long-lasting effect of the acute mental stress induced by the MIST on the STRESS group, who seemed to perceive the VS task as ‘less demanding’ due to the initial stress manipulation, while for controls, VS could be considered the first truly demanding task. In addition, in line with previous studies suggesting that acute stress exposure improves general alertness and cognitive control^56^, our results might indicate that the increased alertness in the STRESS group also reflects a lower need for additional autonomic activation during the attention task.

The current study has some limitations that need to be disclosed and properly discussed. First, since the participants receiving stress manipulation were led to believe they would be rewarded based on their performance, we could not implement a crossover protocol and randomize the order of the proposed tasks. Thus, it could be questioned whether our results were influenced by the time the sensors were worn. However, aside from the tonic level of the EDA, which was excluded from the analysed parameters for this very reason, the other parameters exhibited modulation patterns consistent with the manipulation of the ANS. In particular, parameters principally reflecting sympathetic activation (nSCR, SCR, and AmpSum) were differently modulated by the three tasks, indicating a higher activation for the MIST and VS, while a balanced response was observed for most of the HRV parameters, suggesting an interplay between sympathetic and parasympathetic activation. Still, we cannot entirely exclude the presence of a task sequence effect in our results, which may have influenced the effect size of the differences observed between protocol conditions.

Second, our experimental sample was highly heterogeneous in terms of professional background. Thus, the arithmetic exercises proposed in the MIST task were simpler for participants who were accustomed to working with numbers and more challenging for others, increasing the inter-subject variability in perceived stress, even within groups. This variability may have reduced the possibility of identifying significant differences between the experimental groups. Indeed, some controls verbally reported a highly perceived frustration after the ‘control’ MIST, while some STRESS participants did not respond as expected, either in terms of ANS modulation or according to psychometric indicators. Future studies may include at least two stress-inducing tasks belonging to different psychological domains (e.g., social, cognitive), as proposed in^5,8,9^, to possibly observe more specific stress responses. However, while this limitation could be attributed to the MIST task, our study enabled different ANS responses to be identified, which could help to improve our understanding of the specific nature of stress induced by cognitive tasks. It is also worth mentioning that stress responses may be influenced by individual vulnerability or resilience to stressors, which cannot be estimated a priori without tracing the profile of each participants response at baseline^4^. Therefore, further research aimed at better understanding the effect of acute stress should include a pre-experiment assessment of the included participants to understand their vulnerability/resilience to stressors.

Finally, a third important limitation concerns the collection of cortisol data using saliva samples, which led to a negative result, since no clear modulations in cortisol concentration were detected, in contrast with our hypothesis. A possible reason is related to the short time delay between the task execution and the collection of the cortisol samples, that we based on previous literature^32^, but that, in our case, may have been too short for complete cortisol release, preventing the detection of significant variations in the metabolic response. Therefore, future study designs, including metabolomic sample collection, should consider longer time intervals between tasks and between each task and the collection of the cortisol sample, to ensure that the metabolomic response has been completely activated. As an alternative, other faster biochemical mediators, such as amylase and chromogranin A^57^can be considered. To further interpret our negative results with cortisol data, the relation between salivary cortisol responses and other factors that were not controlled in our protocol but that have been associated with cortisol responses should also be mentioned. Among these, gender, social factors, personality, and personal habits, such as smoking, diet, and alcohol consumption, may influence the individual response to acute stress and the associated metabolic activity^58^. A direct influence is exerted by endogenous sex hormone levels, depending on the phase of the female menstrual cycle^59^, and the circadian rhythm^60^. Therefore, future studies should take into consideration all these factors as much as possible. Specifically, when female individuals of reproductive age are included, the hormonal phase should also be recorded to strengthen the interpretation of metabolic results.

## Conclusion

This study presents a multimodal ANS analysis for characterizing physiological responses to a stimulation protocol based on randomized acute stress manipulation in two groups of healthy adults for a total of 60 participants. Specifically, linear (in both time and frequency domains) and nonlinear HRV indices from ECG, phasic activation features from EDA, and respiratory activity analysis provided an effective characterization of the physiological modulations in response to cognitive tasks under different stress manipulation. Our findings further support the importance of integrating information from respiratory activity for a better interpretation of the frequency-domain analysis of the HRV.

Particularly, the current study illustrates how parameters from various biosignals and physiological domains are modulated by mental stress, supporting the need for multimodal approaches to improve understanding of acute mental stress in practical applications.

## Data Availability

The datasets generated and analysed during the current study could be obtained from the corresponding author on reasonable request.

## References

[1] Tervonen, J. et al. Personalized mental stress detection with self-organizing map: from laboratory to the field. *Comput. Biol. Med.* **124**, (2020). 10.1016/j.compbiomed.2020.103935 32771674

[2] Reali, P., Brugnera, A., Compare, A. & Bianchi, A. M. Efficacy of time- and frequency-domain heart rate variability features in stress detection and their relation with coping strategies. *IFMBE Proc.* vol. 76 209–216 (Springer, 2020).

[3] Vancheri, F., Longo, G., Vancheri, E. & Henein, M. Y. Mental stress and cardiovascular health—part I. *J. Clin. Med.* **11**, 10.3390/jcm11123353 (2022). 10.3390/jcm11123353 PMC9225328 35743423

[4] McEwen, B. S. & Akil, H. Revisiting the stress concept: implications for affective disorders. *J. Neurosci.* **40**, 12–21 (2020). 31896560 10.1523/JNEUROSCI.0733-19.2019 PMC6939488

[5] Immanuel, S., Teferra, M. N., Baumert, M. & Bidargaddi, N. Heart rate variability for evaluating psychological stress changes in healthy adults: A scoping review. *Neuropsychobiology* **82**, 187–202 (2023). 37290411 10.1159/000530376 PMC10614455

[6] Berretz, G., Packheiser, J., Kumsta, R., Wolf, O. T. & Ocklenburg, S. The brain under stress—A systematic review and activation likelihood estimation meta-analysis of changes in BOLD signal associated with acute stress exposure. *Neurosci. Biobehav. Rev.* **124**, 89–99 10.1016/j.neubiorev.2021.01.001 (2021). 10.1016/j.neubiorev.2021.01.001 33497786

[7] Szabo, Y. Z., Slavish, D. C. & Graham-Engeland, J. E. The effect of acute stress on salivary markers of inflammation: A systematic review and meta-analysis. *Brain, Behav. Immun.* **88** 887–900 10.1016/j.bbi.2020.04.078 (2020). 10.1016/j.bbi.2020.04.078 PMC7478864 32371089

[8] Brugnera, A. et al. Heart rate variability during acute psychosocial stress: A randomized cross-over trial of verbal and non-verbal laboratory stressors. *Int. J. Psychophysiol.* **127**, 17–25 (2018). 29501671 10.1016/j.ijpsycho.2018.02.016

[9] Ernst, H. et al. Assessment of the human response to acute mental stress-An overview and a multimodal study. *PLoS One* **18**, (2023). 10.1371/journal.pone.0294069 PMC10635557 37943894

[10] Lucini, D., Norbiato, G., Clerici, M. & Pagani, M. Hemodynamic and autonomic adjustments to real life stress conditions in humans. *Hypertension* **39**, 184–188 (2002). 11799100 10.1161/hy0102.100784

[11] Brugnera, A. et al. Cortical and cardiovascular responses to acute stressors and their relations with psychological distress. *Int. J. Psychophysiol.* **114**, 38–46 (2017). 28174110 10.1016/j.ijpsycho.2017.02.002

[12] Malik, M. Heart rate variability. *Ann. Noninvasive Electrocardiol.* **1**, 151–181 (1996).

[13] Pereira, T., Almeida, P. R., Cunha, J. P. S. & Aguiar, A. Heart rate variability metrics for fine-grained stress level assessment. *Comput. Methods Programs Biomed.* **148**, 71–80 (2017). 28774440 10.1016/j.cmpb.2017.06.018

[14] Castaldo, R., Montesinos, L., Melillo, P., James, C. & Pecchia, L. Ultra-short term HRV features as surrogates of short term HRV: a case study on mental stress detection in real life. *BMC Med. Inf. Decis. Mak.* **19**, 12 (2019). 10.1186/s12911-019-0742-y PMC6335694 30654799

[15] Lucini, D., Di Fede, G., Parati, G. & Pagani, M. Impact of chronic psychosocial stress on autonomic cardiovascular regulation in otherwise healthy subjects. *Hypertension* **46**, 1201–1206 (2005). 16203875 10.1161/01.HYP.0000185147.32385.4b

[16] Hernando, A. et al. Inclusion of respiratory frequency information in heart rate variability analysis for stress assessment. *IEEE J. Biomed. Health Inf.* **20**, 1016–1025 (2016). 10.1109/JBHI.2016.2553578 27093713

[17] Nicolò, A., Massaroni, C., Schena, E. & Sacchetti, M. The importance of respiratory rate monitoring: from healthcare to sport and exercise. *Sensors* **20**, 6396 (2020). 33182463 10.3390/s20216396 PMC7665156

[18] Homma, I. & Masaoka, Y. Breathing rhythms and emotions. *Exp. Physiol.* **93**, 1011–1021 (2008). 18487316 10.1113/expphysiol.2008.042424

[19] Hayano, J. & Yuda, E. Pitfalls of assessment of autonomic function by heart rate variability. *J. Physiol. Anthropol.* **38**, 3 (2019). 30867063 10.1186/s40101-019-0193-2 PMC6416928

[20] Reali, P. et al. Assessing stress variations in children during the strange situation procedure: comparison of three widely used respiratory sinus arrhythmia Estimation methods. *Physiol. Meas.* **42**, 085007 (2021). 10.1088/1361-6579/ac18ff 34325412

[21] Menuet, C. et al. Redefining respiratory sinus arrhythmia as respiratory heart rate variability: an international expert recommendation for terminological clarity. *Nat. Rev. Cardiol.*10.1038/s41569-025-01160-z (2025). 40328963 10.1038/s41569-025-01160-z

[22] Benedek, M. & Kaernbach, C. A continuous measure of phasic electrodermal activity. *J. Neurosci. Methods*. **190**, 80–91 (2010). 20451556 10.1016/j.jneumeth.2010.04.028 PMC2892750

[23] Polo, E. M. et al. Comparative assessment of physiological responses to emotional elicitation by auditory and visual stimuli. *IEEE J. Transl Eng. Health Med.* **12**, 171–181 (2024). 38088996 10.1109/JTEHM.2023.3324249 PMC10712661

[24] Tian, Y. et al. Physiological signal analysis for evaluating flow during playing of computer games of varying difficulty. *Front. Psychol.* **8**, (2017). 10.3389/fpsyg.2017.01121 PMC5495833 28725206

[25] Feng, Y. X., Tang, T. B. & Ho, E. T. W. Phasic electrodermal activity indicates changes in workload and affective states. in *International Conference on Intelligent Cybernetics Technology & Applications (ICICyTA)* 133–137 10.1109/ICICyTA53712.2021.9689112 (IEEE, 2021).

[26] Zhou, Y. et al. Inference-enabled tracking of acute mental stress via multi-modal wearable physiological sensing: A proof-of-concept study. *Biocybern Biomed. Eng.* **44**, 771–781 (2024).

[27] Giorgi, A. et al. Wearable technologies for mental Workload, Stress, and emotional state assessment during Working-Like tasks: A comparison with laboratory technologies. *Sensors* **21**, 2332 (2021). 33810613 10.3390/s21072332 PMC8036989

[28] Rodríguez-Arce, J., Lara-Flores, L., Portillo-Rodríguez, O. & Martínez-Méndez, R. Towards an anxiety and stress recognition system for academic environments based on physiological features. *Comput. Methods Programs Biomed.* **190**, (2020). 10.1016/j.cmpb.2020.105408 32139112

[29] Zhu, L. et al. Stress detection through Wrist-Based electrodermal activity monitoring and machine learning. *IEEE J. Biomed. Health Inf.* **27**, 2155–2165 (2023). 10.1109/JBHI.2023.3239305 37022004

[30] Gedam, S. & Paul, S. A. Review on mental stress detection using wearable sensors and machine learning techniques. *IEEE Access.* **9**, 84045–84066 (2021).

[31] Steffen, P. R. Using the research domain criteria as a framework to integrate psychophysiological findings into stress management and psychotherapy interventions. *Front. Neuroergonom.* **4**, (2023). 10.3389/fnrgo.2023.1245946 PMC10790878 38234487

[32] Dedovic, K. et al. The Montreal imaging stress task: using functional imaging to investigate the effects of perceiving and processing psychosocial stress in the human brain. *J. Psychiatr. Neurosci.* **30** (2005). PMC1197276 16151536

[33] Pruessner, J. C. et al. Deactivation of the limbic system during acute psychosocial stress: evidence from positron emission tomography and functional magnetic resonance imaging studies. *Biol. Psychiatry*. **63**, 234–240 (2008). 17686466 10.1016/j.biopsych.2007.04.041

[34] Tom, S. M., Fox, C. R., Trepel, C. & Poldrack, R. A. The neural basis of loss aversion in Decision-Making under risk. *Sci. (1979)*. **315**, 515–518 (2007). 10.1126/science.1134239 17255512

[35] Chandrasekhar Pammi, V. S. et al. Neural loss aversion differences between depression patients and healthy individuals: A functional MRI investigation. *Neuroradiol. J.* **28**, 97–105 (2015). 25923684 10.1177/1971400915576670 PMC4757155

[36] Pan, J. & Tompkins, W. J. A Real-Time QRS detection algorithm. *IEEE Trans. Biomed. Eng.* **BME-32**, 230–236 (1985). 10.1109/TBME.1985.325532 3997178

[37] Reali, P., Lolatto, R., Coelli, S., Tartaglia, G. & Bianchi, A. M. Information retrieval from photoplethysmographic sensors: A comprehensive comparison of practical interpolation and Breath-Extraction techniques at different sampling rates. *Sensors* **22**, 1428 (2022). 35214329 10.3390/s22041428 PMC8877143

[38] Baselli, G., Porta, A., Rimoldi, O., Pagani, M. & Cerutti, S. Spectral decomposition in multichannel recordings based on multivariate parametric identification. *IEEE Trans. Biomed. Eng.* **44**, 1092–1101 (1997). 9353988 10.1109/10.641336

[39] Faes, L. et al. Information decomposition in the frequency domain: a new framework to study cardiovascular and cardiorespiratory oscillations. *Philosoph. Trans. Royal Soc. A: Math. Phys. Eng. Sci.* **379**, (2021). 10.1098/rsta.2020.0250 34689619

[40] Porta, A. et al. Categorizing the role of respiration in cardiovascular and cerebrovascular variability interactions. *IEEE Trans. Biomed. Eng.* **69**, 2065–2076 (2022). 34905489 10.1109/TBME.2021.3135313

[41] Guede-Fernandez, F., Fernandez-Chimeno, M., Ramos-Castro, J. & Garcia-Gonzalez, M. A. Driver drowsiness detection based on respiratory signal analysis. *IEEE Access.* **7**, 81826–81838 (2019).

[42] Bianchi, A. et al. Spectral analysis of heart rate variability signal and respiration in diabetic subjects. *Med. Biol. Eng. Comput.* **28**, 205–211 (1990). 2377001 10.1007/BF02442668

[43] Cerutti, S. et al. Compressed spectral arrays for the analysis of 24-hr heart rate variability signal: enhancement of parameters and data reduction. *Comput. Biomed. Res.* **22**, 424–441 (1989). 2776446 10.1016/0010-4809(89)90036-0

[44] Faes, L. et al. Causal transfer function analysis to describe closed loop interactions between cardiovascular and cardiorespiratory variability signals. *Biol. Cybern*. **90**, 390–399 (2004). 15278463 10.1007/s00422-004-0488-0

[45] Widjaja, D., Caicedo, A., Vlemincx, E., Van Diest, I. & Van Huffel, S. Separation of respiratory influences from the tachogram: A methodological evaluation. *PLoS One*. **9**, e101713 (2014). 25004139 10.1371/journal.pone.0101713 PMC4086956

[46] Baselli, G., Cerutti, S., Civardi, S., Malliani, A. & Pagani, M. Cardiovascular variability signals: towards the identification of a closed-loop model of the neural control mechanisms. *IEEE Trans. Biomed. Eng.* **35**, 1033–1046 (1988). 3220497 10.1109/10.8688

[47] Hao, T., Zheng, X., Wang, H., Xu, K. & Chen, S. Linear and nonlinear analyses of heart rate variability signals under mental load. *Biomed. Signal. Process. Control*. **77**, 103758 (2022).

[48] Henriques, T. et al. Nonlinear methods most applied to heart-rate time series: A review. *Entropy* **22**10.3390/e22030309 (2020). 10.3390/e22030309 PMC7516766 33286083

[49] Ramshur, J. T. J. & Design evaluation, and application of heart rate variability analysis software (HRVAS) University of Memphis. (2010).

[50] Fritz, C. O., Morris, P. E. & Richler, J. J. Effect size estimates: current use, calculations, and interpretation. *J. Exp. Psychol. Gen.* **141**, 2–18 (2012). 21823805 10.1037/a0024338

[51] Grossman, P. & Taylor, E. W. Toward Understanding respiratory sinus arrhythmia: relations to cardiac vagal tone, evolution and biobehavioral functions. *Biol. Psychol.* **74**, 263–285 (2007). 17081672 10.1016/j.biopsycho.2005.11.014

[52] Ghibaudo, V., Granget, J., Dereli, M., Buonviso, N. & Garcia, S. A Unifying method to study respiratory sinus arrhythmia dynamics implemented in a new toolbox. *eNeuro* **10**, ENEURO.0197-23.2023 (2023). 10.1523/ENEURO.0197-23.2023 PMC10614108 37848290

[53] Rahman, S., Habel, M. & Contrada, R. J. Poincaré plot indices as measures of sympathetic cardiac regulation: responses to psychological stress and associations with pre-ejection period. *Int. J. Psychophysiol.* **133**, 79–90 (2018). 30107195 10.1016/j.ijpsycho.2018.08.005

[54] Lewis, M. J. & Short, A. L. Sample entropy of electrocardiographic RR and QT time-series data during rest and exercise. *Physiol. Meas.* **28**, 731–744 (2007). 17664626 10.1088/0967-3334/28/6/011

[55] Reali, P., Cosentini, C., de Carvalho, P., Traver, V. & Bianchi, A. M. Towards the development of physiological models for emotions evaluation. In *40th Annual International Conference of the IEEE Engineering in Medicine and Biology Society (EMBC)* 110–113 10.1109/EMBC.2018.8512236 (IEEE, 2018). 10.1109/EMBC.2018.8512236 30440353

[56] Qi, M. & Gao, H. Acute psychological stress promotes general alertness and attentional control processes: an ERP study. *Psychophysiology* **57**, (2020). 10.1111/psyp.13521 31898811

[57] Jantaratnotai, N., Rungnapapaisarn, K., Ratanachamnong, P. & Pachimsawat, P. Comparison of salivary cortisol, amylase, and chromogranin A diurnal profiles in healthy volunteers. *Arch. Oral Biol.* **142**, 105516 (2022). 35952574 10.1016/j.archoralbio.2022.105516

[58] Kudielka, B. M., Hellhammer, D. H. & Wüst, S. Why do we respond so differently? Reviewing determinants of human salivary cortisol responses to challenge. *Psychoneuroendocrinology* **34**, 2–18 (2009). 19041187 10.1016/j.psyneuen.2008.10.004

[59] Wolfram, M., Bellingrath, S. & Kudielka, B. M. The cortisol awakening response (CAR) across the female menstrual cycle. *Psychoneuroendocrinology* **36**, 905–912 (2011). 21237574 10.1016/j.psyneuen.2010.12.006

[60] Martel, J. et al. Effects of light, electromagnetic fields and water on biological rhythms. *Biomed. J.* **48**, 100824 (2025). 39672328 10.1016/j.bj.2024.100824 PMC12173616

